# Limbic-predominant age-related TDP-43 encephalopathy neuropathologic change and microvascular pathologies in community-dwelling older persons

**DOI:** 10.1111/bpa.12939

**Published:** 2021-02-23

**Authors:** Sonal Agrawal, Lei Yu, Alifiya Kapasi, Bryan D. James, Konstantinos Arfanakis, Lisa L. Barnes, David A. Bennett, Sukriti Nag, Julie A. Schneider

**Affiliations:** 1 Rush Alzheimer’s Disease Center, Rush University Medical Center, Chicago, IL, USA; 2 Department of Pathology, Rush University Medical Center, Chicago, IL, USA; 3 Department of Neurological Sciences, Rush University Medical Center, Chicago, IL, USA; 4 Department of Internal Medicine, Rush University Medical Center, Chicago, IL, USA; 5 Department of Biomedical Engineering, Illinois Institute of Technology, Chicago, IL, USA; 6 Department of Behavioral Sciences, Rush University Medical Center, Chicago, IL, USA

**Keywords:** arteriolosclerosis, brain watershed regions, cerebrovascular pathology, limbic-predominant age-related TDP-43 encephalopathy

## Abstract

Limbic-predominant age-related transactive response DNA-binding protein 43 (TDP-43) encephalopathy neuropathologic change (LATE-NC) and microvascular pathologies, including microinfarcts, cerebral amyloid angiopathy (CAA), and arteriolosclerosis are common in old age. A relationship between LATE-NC and arteriolosclerosis has been reported in some but not all studies. The objectives of this study were to investigate the frequency of co-occurring LATE-NC and microvascular pathologies and test the hypothesis that arteriolosclerosis, specifically, is related to LATE-NC in brains from community-dwelling older persons. Analyses included 749 deceased participants with completed data on LATE-NC and microvascular pathology from 3 longitudinal clinical pathologic studies of aging. Given the specific interest in arteriolosclerosis, we expanded the examination of arteriolosclerosis to include not only the basal ganglia but also two additional white matter regions from anterior and posterior watershed territories. Ordinal logistic regression models examined the association of microvascular pathology with LATE-NC. LATE-NC was present in 409 (54.6%) decedents, of which 354 (86.5%) had one or multiple microvascular pathologies including 132 (32.3%) with moderate-severe arteriolosclerosis in basal ganglia, 195 (47.6%) in anterior watershed, and 144 (35.2%) in posterior watershed; 170 (41.5%) with moderate-severe CAA, and 150 (36.6%) with microinfarcts. In logistic regression models, only posterior watershed arteriolosclerosis, but not other regions of arteriolosclerosis was associated with a higher odds of more advanced LATE-NC stages (Odds Ratio = 1.12; 95% Confidence Interval = 1.01–1.25) after controlling for demographics, AD, and other age-related pathologies. Capillary CAA, but not the severity of CAA was associated with an increased odds of LATE-NC burden (Odds Ratio = 1.71; 95% Confidence Interval = 1.13–2.58). Findings were unchanged in analyses controlling for APOE ε4, vascular risk factors, or vascular diseases. These findings suggest that LATE-NC with microvascular pathology is a very common mixed pathology and small vessel disease pathology may contribute to LATE-NC in the aging brain.

## 1 | INTRODUCTION

Limbic-predominant age-related transactive response DNA-binding protein 43 (TDP-43) encephalopathy neuropathological change (LATE-NC) is characterized by TDP-43 proteinopathy that primarily affects limbic structures of the brain, with or without coexisting hippocampal sclerosis pathology (1). It is common in the brains of persons over the age of 80 years, and is present in more than 20% of autopsy brains depending on the sample (1). LATE-NC pathology is associated with amnestic dementia that produces clinical features very similar to Alzheimer’s disease-neuropathological changes with prominent impairment in episodic memory (1–4). Neuropathological studies from our group (3,5) and others (6–11) reveal that LATE-NC pathology rarely occurs alone and commonly coexists with Alzheimer’s disease (AD), hippocampal sclerosis (HS), and Lewy body (LB) pathology in older adults. However, little is known about the co-occurrence and relationship of microvascular brain pathology with LATE-NC.

There is increasing recognition in the field of dementia that small vessel disease pathologies and injury are frequent in old age, commonly co-occurs with AD, and are important drivers of late-life cognitive decline and dementia in the aging brain (12–17). While a few neuropathologic studies have found relationships between arteriolosclerosis and pathogenic TDP-43 accumulation or HS (18,19), this relationship could be confounded by other common pathologies in the aging brain. Moreover, the frequency of microvascular pathologies in people with LATE-NC and the extent to which microvascular pathologies are associated with LATE-NC have not been systematically investigated in community-dwelling older persons; therefore, a better understanding of how these two brain pathologies are interrelated could potentially open new avenues for therapeutics of diseases that contribute to dementia in older age.

The overall goal of this study was first to describe the frequency of co-existing LATE-NC and microvascular pathologies and second to determine which microvascular pathologies are associated with LATE-NC. Here, we collected clinical and neuropathological data from three longitudinal clinical-pathologic studies of aging and performed a comprehensive study of LATE-NC and microvascular pathologies. We investigated the frequency of microvascular pathologies [arteriolosclerosis in three regions (anterior watershed, posterior watershed, and basal ganglia), cerebral amyloid angiopathy, and microinfarcts] in people with LATE-NC pathology and tested the hypothesis that microvascular pathologies are associated with LATE-NC, above and beyond demographics, and other age-related neurodegenerative (AD and LB) and cerebrovascular pathologies (atherosclerosis and gross infarcts).

## 2 | MATERIALS AND METHODS

### 2.1 | Participants

Older participants (age 65+) without known dementia were enrolled in three ongoing longitudinal, community-based clinical-pathologic studies of aging and dementia: 1. Religious Orders Study (ROS), 2. The Rush Memory and Aging Project (MAP), and 3. Minority Aging Research Study (MARS) (20,21). Each participant in this analysis consented for annual clinical evaluation and brain donation at the time of death. At the time of analysis, a total of 4425 individuals had been enrolled in these studies (MAP, n = 2184; ROS, n = 1463; MARS, n = 778). Among these participants, 4349 individuals had completed baseline cognitive assessments, 2043 participants died, 1650 were autopsied (total autopsy rate 80.8%). Out of 1650 autopsied brains, anterior white matter watershed and posterior white matter watershed arteriolosclerosis measures were obtained from 763 consecutive autopsy cases of sub-study beginning in 2012. Furthermore, 14 subjects were excluded because of a pathological diagnosis of frontotemporal lobar degeneration-TDP43, atypical tauopathies, or incomplete neuropathology data on LATE-NC or microvascular pathology and a total of 749 participants were included in this study. The mean age at death was 90.3 years (SD = 6.4), mean education was 16 years (SD = 3.5), 69% were females, 24.8% were *APOE* ε4 carriers, 96% white, and 4% black.

### 2.2 | Clinical diagnosis and clinical vascular data

Cognitive tests including a harmonized battery of 19 tests and the Mini-Mental State Examination (MMSE) were assessed annually as previously described (20). After death, all clinical data were reviewed and the final clinical diagnosis of Alzheimer’s dementia was rendered by an expert neurologist blinded to the pathological data as previously described (22). In addition to clinical diagnosis, a variety of clinical data on a range of vascular risk factors (hypertension, diabetes, and smoking) and vascular diseases (stroke, myocardial infarction, and claudication) were collected annually based on the participant’s self-report questions, clinical examinations, and medication inspection, as previously described (23,24). For statistical analyses, composite measures of vascular risk and vascular disease burden proximate to death (scores ranged from 0 to 3 with higher scores reflecting greater burden) were computed as described previously (25).

### 2.3 | Postmortem neuropathological assessment

Brain autopsies, tissue dissection, fixation, sectioning, and a uniform gross and microscopic examination using a standard protocol were performed as described elsewhere (26). The median postmortem interval was 9.7 hours (Interquartile range: 4.8).

### 2.4 | LATE-NC pathology

**FIGURE 1:**
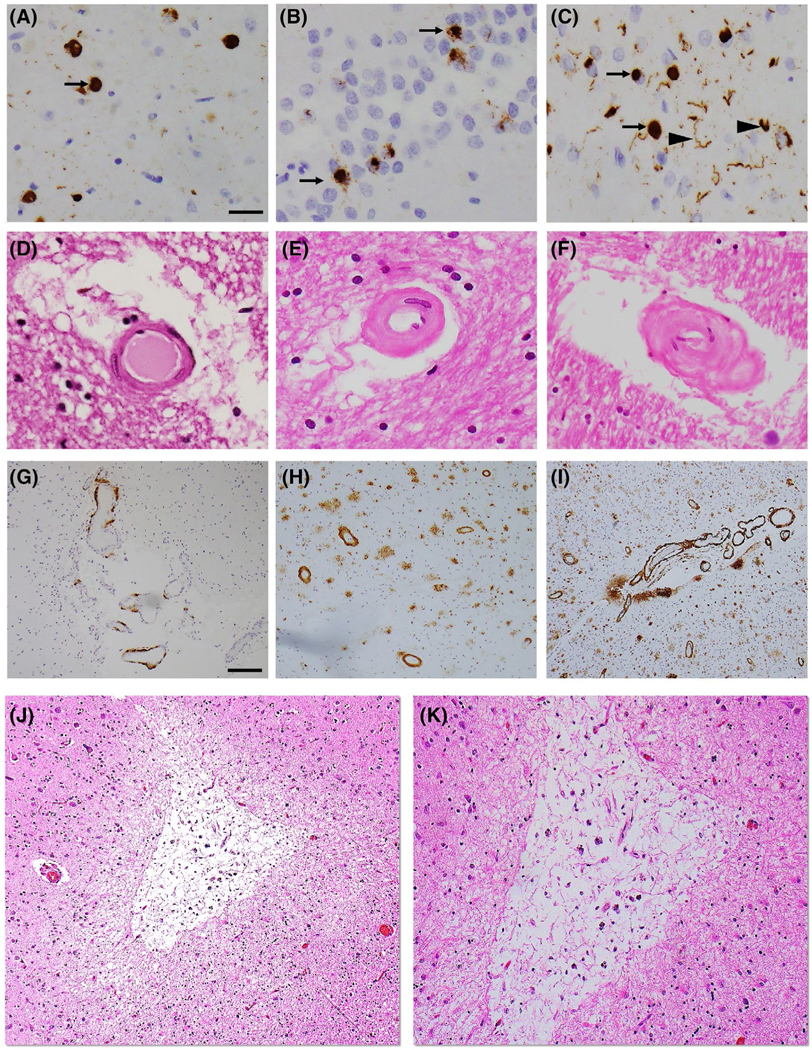
Representative photomicrographs of LATE-NC and microvascular pathologies. TDP-43 immunohistochemical staining of amygdala (A), dentate gyrus, hippocampus (B), and middle frontal gyrus (C) indicates the presence of TDP-43 cytoplasmic inclusions (arrow) with dendritic neurites (arrowhead). H&E staining in the posterior watershed region shows severity of arteriolosclerosis: mild (D), moderate (E), and severe (F). 4G8 immunostaining in the inferior parietal lobule shows severity of amyloid angiopathy: mild (G), moderate (H), and severe (I). Low (J) and high (K) magnification images of a chronic microinfarct in the midfrontal gyrus. Scale bars: 50 μm (A–F), 200 μm (G–J), and 100 μm (K)

LATE-NC pathology was assessed in the amygdala, limbic [entorhinal cortex, hippocampus (dentate gyrus and CA1 sector)], midfrontal, middle temporal, anterior temporal tip, and inferior orbital frontal cortices. Immunohistochemistry with a phosphorylated monoclonal TAR5P-1 D3 anti-TDP-43 antibody (pS409/410; 1:100) was used to detect the abnormal TDP-43 inclusions in the cytoplasm of neurons and glial cells. TDP-43 cytoplasmic inclusions were manually counted in a 0.25 mm^2^ area of greatest density and the presence and severity of TDP-43 cytoplasmic inclusions were rated from 0 (none) to 6 (severe) point scale, as described previously (3). Based on the LATE-NC working group recommendations, we summarized LATE-NC into four distinct stages: stage 0 (absence of TDP-43), stage 1 (localized to amygdala only), stage 2 (extension to the hippocampus or entorhinal cortex), and stage 3 (extension into the neocortex) (1) (Figure 1A–C). F or descriptive purpose, LATE-NC distribution was dichotomized into present or absent where LATE-NC w as defined as present if TDP-43 inclusions were found in any assessed regions and absent if no TDP-43 inclusions were found in any of these regions. In the main analysis, we used four levels of LATE-NC stages (stage 0, stage 1, stage 2, and stage 3).

### 2.5 | Microvascular pathology

#### 2.5.1 | Arteriolosclerosis

Arteriolosclerosis was defined as a fibrohyalinotic thickening of deep penetrating small arterioles with subsequent narrowing of the vascular lumen (Figure 1D–F). We assessed arteriolosclerosis on 6 μm H&E stained sections from three brain regions: basal ganglia, anterior watershed, and posterior watershed using a semi-quantitative grading system from 0 (no arteriolosclerosis) to 6 (severe) in the brain, as described previously (27). The basal ganglia arteriolosclerosis measure was collected at the level of the anterior commissure and included caudate, putamen, and internal capsule. The anterior watershed arteriolosclerosis was collected from the anterior white matter deep to the midfrontal cortex and posterior watershed arteriolosclerosis was taken from the inferior-medial white matter of the posterior parietal cortex. It is important to note that arteriolosclerosis severity was assessed only in the white matter of the two watershed regions. In descriptive analysis, we grouped arteriolosclerosis pathology into two levels (moderate-to-severe vs. none-to-mild) while in the main analysis, we used a 6-point severity scale for all three-regional arteriolosclerosis.

#### 2.5.2 | Cerebral amyloid angiopathy

Cerebral amyloid angiopathy was examined in meningeal and parenchymal vessels from each four brain regions: midfrontal, middle temporal, inferior parietal, and occipital cortices that were immunostained for β-amyloid and scored from 0 to 4, where 0 = no deposition, 1 = scattered segmental but no circumferential deposition, 2 = circumferential deposition up to 10 vessels, 3 = circumferential deposition up to 75% of the region, and 4 = circumferential deposition over 75% of the total region (Figure 1G–I). The CAA score for each region was the maximum of the meningeal and parenchymal CAA scores. Each regional amyloid angiopathy scores were averaged across the four neocortical regions to create a mean score of none, mild, moderate, and severe using cutoffs determined by the neuropathologist. For main analysis, four-level of CAA severity (none, mild, moderate, and severe) was used while a dichotomized variable (moderate-to-severe vs. none-to-mild) was used in the descriptive analysis, as described previously (15). Finally, capillary CAA was assessed as present or absent in four above-mentioned neocortical regions and analyzed as binary variable based on the presence of capillary CAA in any of the regions, as described previously (15).

#### 2.5.3 | Microscopic cerebral infarcts

Microscopic infarcts were identified in 6 μm H&E stained sections of non-watershed cortical regions (anterior cingulate, mid temporal, inferior parietal, and occipital cortices), watershed cortical regions (midfrontal, anterior watershed, and posterior watershed), limbic regions (amygdala, entorhinal cortex, and hippocampus), subcortical regions (basal ganglia and anterior thalamus), midbrain (substantia nigra), and cerebellum including the dentate nucleus, during the microscopic evaluation. Age was noted for each microscopic infarct and only chronic infarctions were included for the analysis as acute and subacute microscopic infarcts may be agonal and mostly related to perimortem events. Finally, we used dichotomous variables rated as present or absent, as described earlier (28,29) (Figure 1J,K).

### 2.6 | Hippocampal sclerosis

Hippocampal sclerosis (HS) was assessed unilaterally in the mid-hippocampus at the level of the lateral geniculate nucleus and used as present or absent for analyses based on the presence of severe neuronal loss and gliosis in the CA1/subiculum sub-region of the hippocampus (3).

### 2.7 | Other neurodegenerative and vascular pathologies

Alzheimer’s disease (AD) pathology such as neuritic plaques, diffuse plaques, and neurofibrillary tangles were assessed from the midfrontal, middle temporal, entorhinal, and inferior parietal cortices, and hippocampus using a modified Bielschowsky silver stain. We used a graticule to count the total number of each AD marker in a 1-mm^2^ of highest density area (x100 magnification). Counting was performed by board-certified neuropathologist or trained technician blinded to all clinical data (30) and interrater reliability on 40 cases was high (r = 0.89–0.93), as previously assessed elsewhere (31).

Since we wanted an overall indicator of the burden of AD pathology with minimal measurement error, we developed global summary measures of AD pathology. In brief, we first converted the raw counts to a standard distribution by dividing each person’s count by the SD for that particular count. Next, we formed summary measures of neuritic plaques, diffuse plaques, and neurofibrillary tangles by averaging the scaled scores for each pathologic index from the five regions. Finally, composite scores of neuritic plaques, diffuse plaques, and neurofibrillary tangles were then averaged to create a global summary measure of AD pathology, as described previously (30). The validity of the global AD pathology summary measure was assessed previously and found to be a reliable method of summarizing the traditional pathologic hallmarks of AD by comparing it with other established methods commonly used to stage and classify AD pathology, including the CERAD system, Braak staging, and NIA Reagan Institute criteria (32–34).

A pathologic diagnosis of AD was determined by intermediate or high likelihood using the modified National Institute on Aging-Reagan criteria (35), and used for descriptive and analysis purposes. In addition, Aβ was detected with immunohistochemistry in eight regions (hippocampus, entorhinal cortex, midfrontal cortex, inferior temporal cortex, angular gyrus, calcarine cortex, anterior cingulate cortex, and superior frontal cortex), quantified the total amyloid load as the mean percent area of cortex occupied by Aβ, and an average composite density value across all brain regions was used as described elsewhere (36).

Lewy body (LB) pathology was evaluated as present and absent from seven brain regions (midfrontal, mid temporal, entorhinal, anterior cingulate, and inferior parietal cortices, amygdala, and substantia nigra) using immunostaining with alpha-synuclein (Zymed LB 509; 1:50; pSyn, 1:20 000; Wako Chemicals). For descriptive and main analysis, a dichotomous variable of LB was used based on the presence of Lewy bodies in any of the region, as described previously (17).

Atherosclerosis was examined by visual inspection of vessels in the Circle of Willis including the vertebral, basilar, posterior cerebral, middle cerebral, and anterior cerebral arteries and their proximal branches with a semi-quantitative grading system from 0 (no atherosclerosis) to 6 (severe atherosclerosis), as previously described (14). For this study, atherosclerosis was grouped into four levels: none, mild, moderate, and severe for main analyses and grouped into two levels (moderate-to-severe vs. none-to-mild) for descriptive analysis.

Cerebral macroscopic infarcts that are visible to the naked eye were collected with their age, volume, side, and location at the time of gross examination. The age of macroscopic infarcts was confirmed by microscopy and only chronic gross infarcts were used in the analysis and evaluated as present or absent, as previously described (26).

### 2.8 | APOE genotyping

APOE genotyping was determined by sequencing rs429358 (codon 112) and rs7412 (codon 158) at exon 4 of the APOE gene as described earlier (37).

### 2.9 | Statistical analysis

We used student *t* and Chi-square tests as appropriate to examine the bivariate relationships between demographics, clinical, and neuropathologic characteristics of the study participants. Pairwise chi-square tests with Bonferroni correction compared the proportions of moderate or severe arteriolosclerosis between three regions.

To test the primary hypothesis, we used ordinal logistic regression analyses to examine the associations of arteriolosclerosis, amyloid angiopathy, and chronic microinfarcts, separately with the odds of having more advanced LATE-NC stages. The core model was adjusted for age at death, sex, and education. Next, we augmented the model by including additional terms for common age-r elated pathologies such as AD pathology score, LB, atherosclerosis, and macroscopic infarcts.

We conducted secondary analyses to assess the robustness of the findings. First, we augmented the model to control for APOE ε4 risk allele, vascular risk burden, and vascular disease burden one at a time. Second, we examined whether the association of posterior watershed arteriolosclerosis with LATE-NC differed by pathologic diagnosis of AD. This was done by adding the interaction term between posterior watershed arteriolosclerosis and pathologic AD. To examine the association between posterior watershed arteriolosclerosis and LATE-NC among people with low amyloid, we repeated the analyses by restricting to subjects with no more than 10^th^ percentile of the amyloid burden.

To examine whether there was association between arteriolosclerosis and HS pathology similar to LATE-NC, we employed separate logistic regression analyses to evaluate the association of arteriolosclerosis with the presence of HS, adjusted for demographics and age-related pathologies.

All analyses were conducted in SAS/STAT version 9.4 (SAS Institute Inc, Cary, NC) using a Hewlett Packard ProLiant ML350 server with LINUX operating system. A nominal threshold of *p* < 0.05 was used for statistical significance, unless otherwise specified.

## 3 | RESULTS

Demographic, clinical, and neuropathologic characteristics of the study participants are shown in Table 1. Microvascular pathologies were very common. Using severity grade of moderate-to-severe for vessel disease and the presence of microinfarcts, about 233 (31%) of persons had basal ganglia arteriolosclerosis, 345 (46%) had anterior watershed arteriolosclerosis, 245 (33%) had posterior watershed arteriolosclerosis, 272 (36%) had CAA, and 274 (37%) had microinfarcts. Of note, 207 (27.6%) of persons had only one microvascular pathology and 423 (56.4%) had more than one microvascular pathology, suggesting that microvascular pathologies commonly co-occur. Specifically, individuals with basal ganglia arteriolosclerosis were more likely to have anterior watershed arteriolosclerosis (*χ*^2^ = 11.9, df = 1, *p* < 0.001) and posterior watershed arteriolosclerosis (*χ*^2^ = 14.9, df = 1, *p* < 0.001). And, those with posterior watershed arteriolosclerosis were more likely to have anterior watershed arteriolosclerosis (*χ*^2^ = 120.1, df = 1, *p* < 0.001). Separately, CAA was correlated with posterior watershed arteriolosclerosis (*χ*^2^ = 5.17, df = 1, *p* = 0.022), but not with other microvascular pathologies. Microscopic infarcts were correlated with basal ganglia arteriolosclerosis (*χ*^2^ = 6.85, df = 1, *p* = 0.008) and posterior watershed arteriolosclerosis (*χ*^2^ = 9.81, df = 1, *p* = 0.001). In comparison of arteriolosclerosis severity between three regions, with Bonferroni correction moderate or severe arteriolosclerosis was more common in anterior watershed (46.1%) than posterior watershed (32.7%) or basal ganglia (31.2%) regions (*p*s < 0.001) suggesting heterogeneity of arteriolosclerosis pathology across the three regions.

Older age was related to all five microvascular pathologies (all five *p*s < 0.01). Also, individuals with microvascular pathologies including basal ganglia arteriolosclerosis, posterior watershed arteriolosclerosis, or CAA, but not anterior watershed arteriolosclerosis or microinfarcts more often had dementia (all three *p*s < 0.001). CAA but no other microvascular pathologies were related to AD pathology (*p* < 0.001).

LATE-NC pathology was present in 409 (54.6%) persons. Out of those, 147 (19.6%) had stage 1, 75 (10%) had stage 2, while 187 (25%) had stage 3 LATE-NC pathology (Table 1). Compared with those without LATE-NC (stage 0), persons with LATE-NC pathology were much older at the time of death; more often had dementia and a lower MMSE score. Over 50% of LATE-NC subjects had dementia (Table 2). All but 15 of the 409 persons with LATE-NC and dementia were also diagnosed with AD or LB pathology. Almost 75% of LATE-NC subjects met the criteria for a pathological diagnosis of AD and almost 31% had LB pathology (Table 2).

Of the 409 participants with LATE-NC, 354 (86.5%) persons had one or more microvascular pathologies in which 132 (32.3%) had moderate-severe arteriolosclerosis in basal ganglia, 195 (47.6%) in anterior watershed, and 144 (35.2%) in posterior watershed, 170 (41.5%) had CAA, and 150 (36.6%) had microinfarcts. Persons with LATE-NC more often had moderate or severe CAA (*χ*^2^ = 10.1, df = 1, *p* = 0.001) (Table 2).

### 3.1 | Association of microvascular pathology with LATE-NC

**FIGURE 2:**
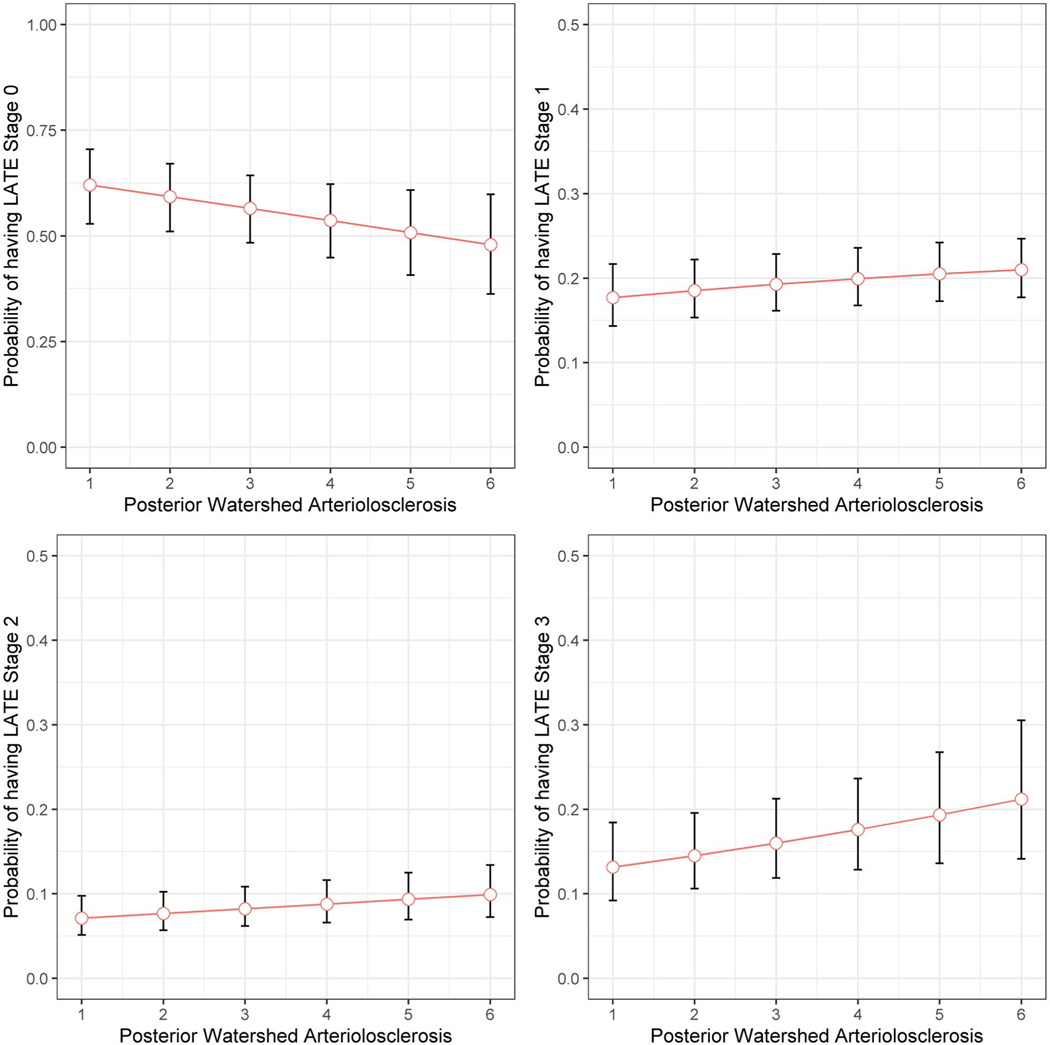
Probability of having LATE-NC stages across the posterior watershed arteriolosclerosis severity. The figure illustrates model predicted probabilities of having LATE-NC for a representative female participant with average age and years of education, as functions of posterior watershed arteriolosclerosis. Red circles indicate model predicted probabilities, and vertical line segments indicate corresponding 95% confidence intervals

We examined the association of each of the microvascular pathologies including arteriolosclerosis in three brain regions, amyloid angiopathy, and microinfarcts with the burden of LATE-NC stages in older persons. Adjusted for age at death, sex, and education, persons with more severe posterior watershed arteriolosclerosis (OR = 1.12; 95%CI: 1.01, 1.24) and separately CAA (OR = 1.26; 95%CI: 1.09, 1.45) had a higher odds of more advanced LATE-NC (Table 3; model 1 and 4). After further adjusting for age-related neurodegenerative (AD and LB) and other vascular (atherosclerosis and gross infarcts) pathologies, only posterior watershed arteriolosclerosis was associated with LATE-NC stages (OR = 1.12; 95%CI: 1.01, 1.25) (Table 3; model 6 and Figure 2). We did not find the association of other microvascular pathologies (basal ganglia arteriolosclerosis, anterior watershed arteriolosclerosis, CAA, or microinfarcts) with LATE-NC stages after controlling for AD, LB, atherosclerosis, and gross infarcts (with all four *p*s > 0.1; data not shown).

We next explored whether the relationship between microvascular pathologies and LATE-NC was affected by other potential confounders. First, since vascular risk factors/diseases may affect the level of microvascular pathologies and LATE-NC (38,39), we repeated our model by including terms for vascular risk burden and vascular disease burden; the association did not change (Table S2; model 2 and 3). Second, APOEε4 was reported to be related to LATE-NC (1) and has a mixed relationship with vascular pathology (40,41). Thus, we controlled the terms for APOE ε4 and the association of posterior watershed arteriolosclerosis and LATE-NC persisted (Table S2; model 4).

Finally, we investigated whether the relationship between posterior watershed arteriolosclerosis and LATE-NC might differ by the presence of pathologic AD diagnosis. We augmented the model by adding an interaction term of posterior watershed arteriolosclerosis and pathologic AD diagnosis. We did not find evidence of an interaction (*p* = 0.205), suggesting the relationship between posterior watershed arteriolosclerosis and LATE-NC did not vary by the pathologic AD diagnosis (data not shown). Next, we examined whether the association of posterior watershed arteriolosclerosis with LATE-NC burden was retained among people with lower amyloid. We did not find an association (parameter estimate = +0.21, *p* = 0.181; data not shown), but this could be caused by smaller sample size (n = 104).

#### 3.1.1 | Additional analyses

Given that capillary CAA and severity of CAA have differential pathologic correlates (15,42), we separately studied the frequency of capillary CAA and repeated the fully adjusted logistic regression model described above after including a term for capillary CAA. Capillary CAA was present in 94 (12%) persons (Table 1). Out of those, 69% had LATE-NC pathology. Unexpectedly, the term for capillary CAA was significantly associated with increase odds of LATE-NC stages (OR = 1.71; 95%CI: 1.13, 2.58; *p* = 0.01) after controlling for demographics, AD, LB, atherosclerosis, and gross infarcts (Table S3; model 1). Furthermore, the finding remained unchanged after adjusted for CAA severity, APOE ε4, vascular risk burden, or vascular disease burden (Table S3; models 2–5).

Because LATE-NC is commonly co-existed with HS pathology and prior works suggested that arteriolosclerosis is specifically related to HS (1,3,19), we separately examined the relationship between arteriolosclerosis and HS pathology. The association was not observed between any regional arteriolosclerosis (basal ganglia, anterior watershed, or posterior watershed) and HS pathology after controlling for demographics and age-related pathologies (with all three *p*s > 0.1; data not shown).

## 4 | DISCUSSION

In this clinicopathologic study of more than 700 older community-dwelling adults, we found that coexisting microvascular and LATE pathologies are very common in the aging brain, with more than 85% of older persons with LATE-NC pathology having one or more microvascular pathologies. Not only do LATE and microvascular pathologies commonly co-occur, subsequent analyses showed that arteriolosclerosis in the posterior watershed region was specifically associated with more severe LATE-NC, after controlling for age at death, AD, and other possible confounders. We also found that CAA specific to capillaries, but not CAA severity was related to LATE-NC burden. These findings underscore the importance of vascular pathologies in relation to LATE and related disorders in older population.

Because LATE-NC is common in advanced age and is associated with cognitive impairment and dementia (1), it is critical to unravel the pathophysiologic mechanisms underlying LATE-NC pathogenesis. Although genetic risk factors such as APOEε4 and neurodegenerative pathologies such as AD and HS have been shown to be associated with an increased likelihood of LATE-NC (1,3,5), less is known about vascular pathologies/injuries which could play a pathophysiologic role in LATE-NC pathogenesis. We are not aware of any previous community-based clinicopathologic study relating the full spectrum of microvascular pathologies to LATE-NC burden. Few studies have explored the association between LATE and large/small vessel disease within specific regions of the brain, and findings are inconsistent. Notably, a recently published study using neuropathology data from the National Alzheimer’s Coordinating Center of 929 persons investigated the association of CAA, arteriolosclerosis, microinfarcts, macroinfarcts, and atherosclerosis pathologies with TDP-43 burden in three different brain regions (18) and found a significant association between moderate-to-severe arteriolosclerosis and TDP-43 pathology present in the entorhinal cortex and inferior temporal cortex. This important study was notable for a large number of subjects and controlling for age and AD pathology but was not community-based and did not control for some of the other vascular pathologies or other possible confounders. Another study using clinic-pathologic data from 23 older persons examined the relationship between macroscopic infarcts and TDP-43 burden and found a significant relationship between macroscopic infarcts and pathogenic TDP-43 accumulation of the basal nucleus of Meynert (43); but this study focused on macroscopic infarcts and examined a small number of cases. Another study from Neurological tissue bank of the Biobank-Hospital Clínic-Institut d’Investigacions Biomèdiques August Pi i Sunyero examined 3three postmortem poststroke brain tissue autopsied at 1–5 days after stroke for TDP-43 expression patterns in the frontal cortex and found increase in the cytoplasmic TDP-43 immunoreactivity (44). Although in our previous study of community-dwelling older persons without pathologic diagnosis of AD and FTLD, the frequency of basal ganglia arteriolosclerosis was reported to be higher in persons with TDP-43 compared to those without TDP-43 (45); however, regression analyses were not done to establish an association between arteriolosclerosis and TDP-43 pathology. Here, we extended our previous studies by investigating arteriolosclerosis and microinfarcts in two additional brain regions (anterior watershed and posterior watershed) with other common brain regions and found that microvascular pathology with LATE-NC is a common mixed pathology, present in 47% (354/749) of brains of older persons. Furthermore, posterior watershed white matter arteriolosclerosis and capillary CAA increased the odds of more severe LATE-NC, after controlling for demographics, gross infarcts, atherosclerosis, AD, and LB pathology. This finding was robust and remained significant when controlling for APOEε4, vascular risk factors, and vascular diseases. Furthermore, our data did not reveal a significant association between arteriolosclerosis and HS pathology. However, the number of cases in HS group was very small, and further study of the role of vascular disease in HS is warranted.

There are three additional important findings in this study to note. First, moderate-to-severe arteriolosclerosis was more common in anterior watershed compared to posterior watershed white matter regions or basal ganglia in the brain, supporting the notion that there is heterogeneity within arteriolosclerosis pathology across brain regions (46). Notably, other studies conducted in our cohorts have also supported the idea of heterogeneity of microvascular pathologies in the brain and indicate the region-specific associations of vascular pathology with neurodegenerative pathology and neurologic outcomes. One study showed that spinal cord arteriolosclerosis but not brain arteriolosclerosis was associated with Parkinsonism (27) as well as another study reported that the frequency of microinfarcts in the cortical watershed regions was much higher compared to other brain regions and specifically associated with cognitive impairment (29). Second, posterior watershed white matter arteriolosclerosis but not basal ganglia or anterior watershed arteriolosclerosis was specifically related to LATE-NC stages increments. There are at least two possible explanations for this region-specific association. First, the posterior watershed region shares the boundary with the precuneus which possesses a significant heavy amyloid plaque burden in AD brains and is considered to be one of the first brain regions to be affected in early AD, which may make this particular region much more important and vulnerable to increase the burden of LATE-NC pathology (47); as studies have indicated the interaction of LATE-NC pathology (presence of TDP-43 inclusions) with neurodegenerative changes in AD (1). However, the association findings in our study persist after adjusting for a pathologic diagnosis of AD. Furthermore, we present data where we examine the association between posterior watershed arteriolosclerosis and LATE-NC in a subsample with low amyloid and we did not find a significant association, but this could be caused by smaller sample size, and future study is warranted. Second, predominant reduced cerebral blood flow and lower perfusion across the posterior watershed areas (precuneus and posterior cingulate gyrus) compared to other brain regions (anterior watershed region and basal ganglia), perhaps distal posterior cerebral arteries, could make this region more susceptible for contributing to neuronal dysfunction in more limbic/temporal regions where LATE-NC preferentially tends to accumulate when small vessel disease coexisted (48,49). This possible explanation is supported by multiple studies demonstrating that vascular dysfunction/small vessel disease and reduced cerebral blood flow in the posterior cingulate cortex and precuneus regions lead to the initiation and aggravation of AD pathology in the medial temporal cortex as well as relate with HS pathology (19,48,50–52). Finally, these finding support the notion that small vessels disease in the brain regions most closely associated with reduced cerebral blood flow and hypoperfusion initiate and contribute to pathophysiologic process of neurodegenerative diseases. However, further studies are needed to examine the factors and underlying mechanism important for the posterior watershed arteriolosclerosis severity in the brain. Importantly we suggest the posterior watershed region is an important region to investigate when studying microvascular pathologies and LATE-NC in the older brain. Finally, capillary CAA, but not the severity of CAA was associated with increased burden of LATE-NC. As small vessels and capillaries are the major route for the perivascular drainage for the elimination of Aβ or interstitial fluid in older individuals, therefore, blockage of drainage pathway by capillary CAA could also be a significant factor in the formation and spread of age-related neurodegenerative proteins including LATE-NC in the brain (42). Although examination of the relationship between capillary CAA and LATE-NC was not the primary hypothesis of this study, future work is necessary to promote our understanding of capillary CAA and its role in the pathophysiology of LATE disease.

The basis for the association of arteriolosclerosis or capillary CAA with LATE-NC is unclear. There has been a long history of studying the association of vascular and neurodegenerative diseases in the aging brain, especially AD pathology. Here we considered and excluded AD pathology and APOEε4 as confounders, and did not find a confounding role for AD pathology or APOEε4; however, given the frequency of these mixed pathologies and association findings from previous autopsy studies on microvascular and AD pathology (15,19,53–55), further study of other common unmeasured factors that may link vascular and neurodegenerative diseases, such as white matter changes, vascular clearance mechanisms, blood–brain barrier disruption, inflammation, oxidative stress, and other possible mechanisms are warranted (56–59).

There are several limitations to this study. First, despite the fact that the data came from the large numbers of community-based participants, the number of minorities included is small (4% of total participants) and further study will be needed in minority cohorts. Second, several microvascular pathologies are measured in the postmortem brain, however, other microvascular pathologies including white matter changes, enlarged perivascular spaces, and microscopic hemorrhages were not assessed in this study. A final limitation is that the current study was not able to depict the underlying mechanism of association of arteriolosclerosis severity or capillary CAA with the odds of LATE-NC in older people. Regardless of limitations, this study has multiple strengths. First, the study has a very high follow-up and autopsy rate, which contributes to internal validity. Second, our study was conducted using a community-based design, rather than an autopsy series or clinic-based setting, which reduces selection bias. Third, we used a uniform neuropathological data collection method to evaluate pathologies associated with age-related neurodegenerative and cerebrovascular diseases. Finally, we assessed microvascular data from multiple brain regions, and provide important and new information regarding small vessel disease with LATE-NC burden.

In conclusion, these data confirm and extend existing literature, showing the high frequency and independent association of small vessel disease, specifically arteriolosclerosis and capillary CAA, with LATE-NC in the aging brain with and without AD. Further study of microvascular pathology in LATE-NC specifically, and neurodegenerative diseases in general is warranted to determine risk factors, clinical consequences, and pathophysiologic mechanisms that can lead to effective treatments of Alzheimer’s and AD-related disorders.

## Supplementary Material

Supplementary material

## Figures and Tables

**TABLE 1:** Basic characteristics of the study participants (n = 749)

Characteristics	Mean (SD) or N (%)
*Demographics*	
Age at death, mean (SD) years	90.3 (6.4)
Female, n (%)	515 (69)
Education, mean (SD) years	16 (3.5)
*Clinical*	
AD Dementia, n (%)	331 (44.2)
^a^APOE ε4, n (%)	186 (25.3)
*Vascular and neuropathologic*	
AD (NIA-Reagan), n (%)	499 (66.6)
LATE-NC stages, n (%)	
Stage 0	340 (45.4)
Stage 1	147 (19.6)
Stage 2	75 (10)
Stage 3	187 (25)
Hippocampal sclerosis	70 (9.35)
AWS arteriolosclerosis^e^, n (%)	345 (46.1)
^b^BG arteriolosclerosis^e^, n (%)	233 (31.1)
PWS arteriolosclerosis^e^, n (%)	245 (32.7)
^c^CAA^e^, n (%)	272 (36.5)
^d^Capillary CAA, n (%)	94 (12.5)
Chronic microinfarcts, n (%)	274 (36.6)

Abbreviations: PWS, posterior watershed; APOE, apolipoprotein; AWS, anterior watershed; BG, basal ganglia; CAA, cerebral amyloid angiopathy; LATE-NC, limbic predominant age-related TDP-43 encephalopathy neuropathological change.

a Data missing for 14 participants.

b Data missing for 1 participant.

c Data missing for 3 participants.

d Data missing for 6 participants.

e Grade of moderate-to-severe

**TABLE 2:** Demographic, clinical, and pathologic characteristics of subjects with and without LATE-NC

Characteristics	No LATE-NC (n = 340)	LATE-NC (n = 409)	*p*-values*	Stage 1 (n = 147)	Stage 2 (n = 75)	Stage 3 (n = 187)
*Demographics*						
Age at death, mean (SD) years	88.8 (6.6)	91.4 (5.9)	<0.001	90.5 (6.4)	91.3 (5.7)	92.2 (5.4)
Female, n (%)	225 (66.1)	290 (70.9)	0.164	96 (65.3)	58 (77.3)	136 (72.7)
Education, mean (SD) years	16.2 (3.7)	15.8 (3.3)	0.121	15.7 (3.3)	16 (3.8)	15.9 (3.2)
*Clinical*						
MMSE score, mean (SD)	22.8 (8)	18.1 (9.8)	<0.001	20.3 (9.5)	19.7 (9.4)	15.8 (9.9)
AD Dementia, n (%)	110 (32.3)	221 (54.1)	<0.001	57 (39)	35 (46.6)	129 (68.9)
^a^APOEε4, n (%)	62 (18.5)	124 (31)	<0.001	36 (25)	21 (28.4)	67 (36.8)
Vascular risk factors burden, mean (SD)	1.2 (0.9)	1.1 (0.8)	0.546	1.1 (0.8)	1.1 (0.8)	1.1 (0.8)
Vascular disease burden, mean (SD)	0.7 (0.8)	0.7 (0.8)	0.343	0.7 (0.8)	0.8 (0.9)	0.6 (0.8)
*Vascular and neuropathologic*						
AWS arteriolosclerosis^d^, n (%)	150 (44.1)	195 (47.6)	0.330	70 (47.6)	29 (38.7)	96 (51.3)
^b^BG arteriolosclerosis^d^, n (%)	101 (29.7)	132 (32.3)	0.436	47 (32.2)	21 (28)	64 (34.2)
PWS arteriolosclerosis^d^, n (%)	101 (29.7)	144 (35.2)	0.110	48 (32.6)	23 (30.7)	73 (39)
^c^CAA^d^, n (%)	102 (30.3)	170 (41.5)	0.001	54 (36.7)	28 (37.3)	88 (47.1)
Microinfarcts, n (%)	124 (36.5)	150 (36.6)	0.953	59 (40.1)	21 (28)	70 (37.4)
Atherosclerosis^d^, n (%)	79 (23.2)	99 (24.2)	0.756	36 (24.4)	18 (24)	34 (18.1)
Macroinfarcts, n (%)	132 (38.8)	133 (32.5)	0.072	46 (31.2)	15 (20)	72 (38.5)
AD (NIA-Reagan), n (%)	196 (57.6)	303 (74.1)	<0.001	97 (65.9)	48 (64)	158 (84.4)
AD pathology score, mean (SD)	0.6 (0.5)	0.8 (0.6)	<0.001	0.7 (0.6)	0.8 (0.7)	0.9 (0.6)
Lewy bodies, n (%)	71 (20.8)	125 (30.6)	0.002	43 (29.2)	27 (36.4)	55 (29.4)
Hippocampal sclerosis, n (%)	3 (0.9)	67 (16.4)	<0.001	3 (2)	7 (9.3)	57 (30.5)

Abbreviations: AD, Alzheimer’s Disease; PWS, posterior watershed; APOE, apolipoprotein; AWS, anterior watershed; BG, basal ganglia; CAA, cerebral amyloid angiopathy; LATE-NC, limbic predominant age-related TDP-43 encephalopathy neuropathological change; MMSE, Mini-Mental-State-Examination.

a Data missing for 14 participants.

b Data missing for 1 participant.

c Data missing for 3 participants.

d Grade of moderate-to-severe.

* *p*-values are comparing persons with and without LATE-NC (i.e., stage 0 vs. stage 1, stage 2, and stage 3)

**TABLE 3:** Association of microvascular pathology with LATE-NC (Odds ratios and 95% confidence intervals, *p*-values)

Predictors	Model 1	Model 2	Model 3	Model 4	Model 5	Model 6*
Age at death	1.06 (1.03, 1.08), *p* < 0.001	1.06 (1.04, 1.09), *p* < 0.001	1.06 (1.04, 1.09), *p* < 0.001	1.06 (1.03, 1.08), *p* < 0.001	1.06 (1.04, 1.09), *p* < 0.001	1.06 (1.04, 1.09), *p* < 0.001
Male sex	0.83 (0.62, 1.12), *p* = 0.241	0.85 (0.63, 1.14), *p* = 0.280	0.86 (0.64, 1.16), *p* = 0.347	0.84 (0.62, 1.13), *p* = 0.268	0.85 (0.63, 1.15), *p* = 0.309	0.92 (0.68, 1.25), *p* = 0.627
Education	0.98 (0.95, 1.02), *p* = 0.539	0.98 (0.95, 1.02), *p* = 0.535	0.98 (0.95, 1.02), *p* = 0.592	0.98 (0.95, 1.02), *p* = 0.555	0.98 (0.94, 1.02), *p* = 0.491	0.99 (0.95, 1.03), *p* = 0.671
PWS arteriolosclerosis	1.11 (1.01, 1.24), *p* = 0.035					1.12 (1.01, 1.25), *p* = 0.038
AWS arteriolosclerosis		1.01 (0.90, 1.12), *p* = 0.882				
BG arteriolosclerosis			0.99 (0.86, 1.13), *p* = 0.924			
CAA				1.26 (1.09, 1.45), *p* < 0.001		
Microinfarcts					0.86 (0.65, 1.14), *p* = 0.308	
Global AD score						1.88 (1.50, 2.35), *p* < 0.001

Abbreviations: PWS, posterior watershed; AWS, anterior watershed; BG, basal ganglia; CAA, cerebral amyloid angiopathy.

* Additionally, adjusted for Lewy body disease, gross infarcts, and atherosclerosis.
