# Supplementary material for: Limbic-predominant age-related TDP-43 encephalopathy neuropathologic change and microvascular pathologies in community-dwelling older persons
Source: Brain Pathol. Author manuscript; Available in PMC 2021 Aug 13. (PMC8363209; doi:10.1111/bpa.12939)
Supplement: Supplementary material [file NIHMS1725329-supplement-Supplementary_material.docx]

Supplementary table:

Table 1: Frequency of microvascular brain pathologies in study analysis

| Microvascular pathology | Descriptive analysis | Number of subjects | Main analysis | Number of subjects |
| --- | --- | --- | --- | --- |
| AWS arteriolosclerosis | None -mild | 404 | None | 19 |
|  |  |  | Mild | 192 |
|  |  |  | Mild-moderate | 193 |
|  | Moderate-severe | 345 | Moderate | 187 |
|  |  |  | Moderate-severe | 119 |
|  |  |  | Severe | 39 |
| BG arteriolosclerosis | None-mild | 515 | None | 36 |
|  |  |  | Mild | 224 |
|  |  |  | Mild-moderate | 255 |
|  | Moderate-severe | 233 | Moderate | 190 |
|  |  |  | Moderate-severe | 39 |
|  |  |  | Severe | 4 |
| PWS arteriolosclerosis | None-mild | 504 | None | 46 |
|  |  |  | Mild | 264 |
|  |  |  | Mild-moderate | 194 |
|  | Moderate-severe | 245 | Moderate | 119 |
|  |  |  | Moderate-severe | 91 |
|  |  |  | Severe | 35 |
| CAA | None-mild | 474 | None | 169 |
|  |  |  | Mild | 305 |
|  | Moderate-severe | 272 | Moderate | 166 |
|  |  |  | Severe | 106 |
| Microinfarcts | Absent | 475 | Absent | 475 |
|  | Present | 274 | Present | 274 |

Table 2: Association of posterior watershed arteriolosclerosis with LATE-NC, after controlling confounders.

| Predictors | Model 1* | Model 2* | Model 3* | Model 4* |
| --- | --- | --- | --- | --- |
| Age-at-death | 1.06 (1.04, 1.09), p<0.001 | 1.06 (1.04, 1.09), p<0.001 | 1.06 (1.04, 1.09), p<0.001 | 1.07 (1.04, 1.09), p<0.001 |
| Male sex | 0.92 (0.68, 1.25), p=0.627 | 0.91 (0.67, 1.23), p= 0.549 | 0.91 (0.67, 1.23), p= 0.553 | 0.92 (0.67, 1.25), p= 0.595 |
| Education | 0.99 (0.95, 1.03), p=0.671 | 0.99 (0.95, 1.03), p=0.737 | 0.99 (0.95, 1.03), p=0.728 | 0.98 (0.94, 1.03), p=0.540 |
| PWS arteriolosclerosis | 1.12 (1.01, 1.25), p=0.038 | 1.12 (1.01, 1.25), p=0.033 | 1.12 (1.01, 1.25), p=0.033 | 1.13 (1.01, 1.26), p=0.026 |
| Vascular risk factors burden |  | 1.02 (0.86, 1.21), p=0.761 |  |  |
| Vascular disease burden |  |  | 0.93 (0.78, 1.10), p=0.423 |  |
| APOE ε4 |  |  |  | 1.83 (1.31, 2.56), p<0.001 |
| Global AD score | 1.88 (1.50, 2.35), p<0.001 | 1.88 (1.50, 2.36), p<0.001 | 1.88 (1.50, 2.35), p<0.001 | 1.68 (1.32, 2.13), p<0.001 |

*Additionally, adjusted for Lewy body disease, gross infarcts, and atherosclerosis.

Abbreviations: AWS, anterior watershed; BG, basal ganglia; CAA, cerebral amyloid angiopathy; PWS, posterior watershed

Table 3: Association of capillary CAA with LATE-NC, after controlling confounders.

| Predictors | Model 1* | Model 2* | Model 3* | Model 4* | Model 5* |
| --- | --- | --- | --- | --- | --- |
| Age-at-death | 1.06 (1.04, 1.09), p<0.001 | 1.06 (1.04, 1.09), p<0.001 | 1.06 (1.04, 1.09), p<0.001 | 1.06 (1.04, 1.09), p<0.001 | 1.07 (1.04, 1.09), p<0.001 |
| Male sex | 0.91 (0.67, 1.23), p=0.563 | 0.90 (0.67, 1.23), p=0.540 | 0.89 (0.66, 1.21), p=0.484 | 0.89 (0.66, 1.21), p=0.495 | 0.91 (0.67, 1.24), p=0.572 |
| Education | 0.99 (0.95, 1.03), p=0.725 | 0.99 (0.95, 1.03), p=0.712 | 0.99 (0.95, 1.03), p=0.810 | 0.99 (0.95, 1.03), p=0.779 | 0.99 (0.95, 1.03), p=0.629 |
| Capillary CAA | 1.71 (1.13, 2.58), p=0.010 | 1.62 (1.04, 2.52), p=0.031 | 1.73 (1.14, 2.61), p=0.008 | 1.71 (1.13, 2.58), p=0.009 | 1.57 (1.03, 2.41), p=0.035 |
| CAA |  | 1.05 (0.89, 1.24), p=0.512 |  |  |  |
| Vascular risk factors burden |  |  | 1.04 (0.88, 1.22), p=0.635 |  |  |
| Vascular disease burden |  |  |  | 0.93 (0.78, 1.11), p=0.44 |  |
| APOE ε4 |  |  |  |  | 1.67 (1.18, 2.35), p=0.003 |
| Global AD score | 1.78 (1.41, 2.24), p<0.001 | 1.74 (1.37, 2.21), p<0.001 | 1.78 (1.42, 2.24), p<0.001 | 1.78 (1.41, 2.24), p<0.001 | 1.63 (1.28, 2.08), p<0.001 |

*Additionally, adjusted for Lewy body, gross infarcts, and atherosclerosis.
